# Male spondyloarthritis patients and those with longer disease duration have less severe disc degeneration: propensity score-matched comparison

**DOI:** 10.1093/rap/rkae015

**Published:** 2024-02-06

**Authors:** Samuel Tin Yan Cheung, Helen Hoi Lun Tsang, Prudence Wing Hang Cheung, Jason Pui Yin Cheung

**Affiliations:** Department of Orthopaedics and Traumatology, The University of Hong Kong, Hong Kong SAR, China; Department of Orthopaedics and Traumatology, The University of Hong Kong, Hong Kong SAR, China; Department of Orthopaedics and Traumatology, The University of Hong Kong, Hong Kong SAR, China; Department of Orthopaedics and Traumatology, The University of Hong Kong, Hong Kong SAR, China

**Keywords:** spondyloarthritis, disc degeneration, modified Stoke Ankylosing Spondylitis Spinal Score, Spondyloarthritis Research Consortium of Canada, MRI

## Abstract

**Objective:**

Using whole spine sagittal T2 MRI, we aimed to compare the severity and prevalence of disc degeneration (DD) in axial SpA patients *vs* the general population and to determine any association between spinal inflammation, structural changes, mobility and DD among SpA patients.

**Methods:**

Two prospectively collected cohorts of SpA patients (*n* = 411) and the general population (*n* = 2007) were recruited. Eventually, 967 participants from the populational cohort and 304 participants from the SpA cohort were analysed. Two hundred and nineteen matched pairs were generated by propensity score matching. Imaging parameters, including Pfirrmann grading, disc herniation, high-intensity zone, Schmorl’s node, Modic change and anterior marrow change were studied and compared from C2/3 to L5/S1. DD was defined as Pfirrmann grade 4 or 5. Demographic factors, including age, sex and BMI, were collected. Multivariable linear regression was used to determine the association between spinal inflammation [Spondyloarthritis Research Consortium of Canada (SPARCC) spine MRI index], structural changes [modified Stoke Ankylosing Spondylitis Spinal Score (mSASSS)] and mobility (BASMI) with lumbar Pfirrmann score.

**Results:**

SpA patients had lower prevalence of DD (*P* < 0.001). The disease stage-stratified regression model showed that SPARCC spinal MRI index was associated with higher lumbar Pfirrmann scores in early disease (β = 0.196, *P* = 0.044), whereas mSASSS was associated with lower lumbar Pfirrmann scores in later disease (β = −0.138, *P* = 0.038). Males had higher mSASSS (*P* < 0.001) and lower odds of whole spine DD (odds ratio = 0.622, *P* = 0.028).

**Conclusion:**

SpA patients had lower DD severity than the general population. Males had higher mSASSSs, and increased mSASSS at later disease was associated with less severe DD.

Key messages

- SpA patients have a lower prevalence of disc degeneration compared with the general population.
- Male SpA patients and those with longer disease duration have less severe disc degeneration.
- Structural change (mSASSS) is associated with lower lumbar Pfirrmann scores in later disease.

## Introduction

Low back pain is the leading cause of disability worldwide [1]. For most individuals, it is impossible to identify a single cause of the pain [1, 2]. Among all known pathological causes of low back pain, age-related disc degeneration (DD) is highly prevalent in the population and is associated with low back pain [3]. Another well-characterized cause of back pain is axial SpA, which is a disease mainly characterized by inflammatory back pain, with or without involvement of the peripheral joints and extramusculoskeletal manifestations [4]. An inter-cohort comparison between SpA and chronic low back pain patients showed that SpA patients, despite having an earlier age of onset of back pain, had better health-related quality of life outcomes, potentially owing to better established treatment guidelines, including the use of NSAIDs as first-line medication [5].

Sagittal MRI of the spine has been shown to have additional diagnostic value in people without sacroiliitis on MRI or radiograph [6], because the consensus statement issued by Assessment in SpondyloArthritis international Society in 2010 [7]. In a French cohort of patients, it has been reported that degenerative changes can be fairly well distinguished from SpA lesions on MRI [8]; however, de Bruin *et al.* [8] found no significant difference in the prevalence of degenerative changes between those fulfilling the Assessment of SpondyloArthritis international Society axial SpA criteria compared with those who did not. There is no previous study looking at the prevalence of DD among SpA patients and its comparison with the general population. This will help our understanding of the risk factors and predictive factors for DD, and can allow us to stratify the cause of the pain and guide the correct management of SpA patients. We hypothesized that SpA disease factors, such as spinal inflammation and structural changes, would have opposing effects on the severity of DD, compared with the general population.

The aim of this study was to compare the severity and prevalence of DD in people diagnosed with axial SpA *vs* the general population, to determine the association between SpA disease duration and sex and the presence of DD, and to look for any association between spinal inflammation, structural changes and mobility with the severity of low back DD.

## Methods

**Figure 1.**
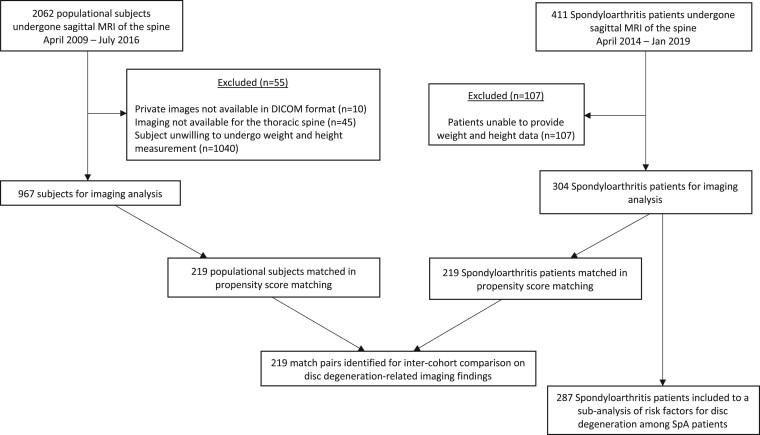
Flowchart of participants included in the study

This study comprised of two longitudinal cohorts. The populational cohort consisted of 2007 participants who were openly recruited via newspaper advertisement, posters and emails, regardless of social and economic status. None had previous spinal surgery, spinal tumours or marked spinal deformities. The selection was not based on the presence or absence of clinical symptoms. The SpA cohort consisted of 411 participants >18 years of age, who were diagnosed to have axial SpA by a rheumatologist. All participants in both cohorts underwent whole spine sagittal T2-weighted MRI. Participants were included into the analysis only if they had complete demographic information, including height and weight. In the end, 967 participants from the populational cohort and 287 participants from the SpA cohort were included in the analysis (Fig. 1). Written informed consent was obtained for each patient, and ethics were approved by the local institutional review board (HKU/HA HKW IRB, IRB reference number: UW15-598), with written patient’s/parent’s informed consent gained. Each author certifies that his or her institution approved the human protocol for this investigation and that all investigations were conducted in conformity with ethical principles of research.

### MRI protocol

3 Tesla High Definition MRI machines were used, and all patients were placed in the supine position for scanning. The following protocol was used: For T2-weighted sagittal scans; field of view of 28 cm × 28 cm, slice thickness of 5 mm, slice spacing of 1 mm, and imaging matrix of 448 × 336. The repetition time was 3320 ms, and the echo time was 85 ms.

### Imaging parameters under study

Disc and endplate measurements included any disc herniation [9], Pfirrmann grading [10], Schmorl’s node [11], high-intensity zone lesions [12] and Modic change [13]. All parameters were studied per level from C2/3 to L5/S1. Disc herniation score had five categories: 0 = no disc herniation; 1 = posterior disc bulging (disc is displaced beyond a virtual line connecting the posterior edges of two adjacent vertebrae); 2 = disc protrusion (distance between the edges of the disc material beyond the disc space is less than the distance between the edges of the base in the same plane); 3 = disc extrusion (distance between the edge of the protruded disc into the spinal canal is greater than the distance between edges of the base of the disc); and 4 = disc sequestration [13]. Schmorl’s node was described as herniation of the nucleus pulposus into the adjacent vertebrae [11]. The high-intensity zone was characterized by a high-intensity area of the annulus fibrosus either posteriorly or anteriorly [12]. Modic changes were changes in signal intensity in the vertebral body adjacent to the endplates [13]. We also noted the anterior location of the Modic change if it occurred in the anterior corner region of the endplate in isolation [14].

For the axial SpA patients, active disease activity was determined using the SPARCC spinal MRI index, which was calculated based on the presence of bone marrow oedema and the intensity and depth of their signals, with a maximum score of 108 [15]. The modified Stoke Ankylosing Spondylitis Spine Score (mSASSS) was used to assess the degree of structural damage, based on the number and severity of erosions, sclerosis, syndesmophytes and ankyloses on the lateral view radiographs of the cervical and lumbosacral spine [16]. Spinal mobility in axial SpA patients was evaluated using BASMI, which is a simple assessment tool based on five measurements that can be performed in the clinical setting [17]. A higher BASMI score indicates worse spinal mobility.

### Clinical assessment

Information related to participant demographics, such as age, sex, weight, height and history of smoking, was collected. Smoking status was categorized as current or previous cigarette smoker, and the duration of smoking (in years) was also considered. Information relating to participants’ back pain was collected in both cohorts, including presence of current back pain, age at onset of back pain and duration of back pain. BMI categories modified for Asian populations based on World Health Organization guidelines were used [18]. Individuals with a BMI of <18.5 kg/m^2^ were classified as underweight, those with a BMI of 18.5–23.0 kg/m^2^ as normal, those with a BMI of 23.0–27.5 kg/m^2^ as overweight, and those with a BMI of >27.5 kg/m^2^ as obese. To assess SpA disease activity, self-assessment questionnaires of BASDAI and patient global assessment were completed, which were used to calculate the AS DAS (ASDAS)-CRP and ASDAS-ESR, respectively. To assess spinal mobility, BASMI was measured independently by one investigator using the linear definition from 0 to 10, with 10 representing the greatest impairment of spinal mobility. All independent scorers were blinded to clinical and MRI data.

### Statistical analysis

Descriptive statistics on patient demographics and frequency statistics on imaging phenotypes were obtained. Pfirrmann grade 4 or 5 was considered as DD [19–21]. The Kolmogorov–Smirnov test was used for normality testing, whereby all continuous variables investigated were significantly different from the normal distribution (*P* < 0.001), hence continuous variables were analysed using non-parametric measures. Given that the baseline demographic variables (Table 1 and Supplementary Table S1, available at *Rheumatology Advances in Practice* online) showed significant differences between the SpA cohort and the population cohort, such as in age, which is a widely recognized factor for DD, we adopted the approach of propensity score matching to allow valid comparisons between the two cohorts.

**Table 1.** Patient characteristics before and after propensity score matching

	Before matching			After matching		
Characteristic	SpA	Population	*P*-value	SpA	Population	*P*-value
Age (mean ± s.d.), years	44.8 ± 12.9	51.4 ± 8.1	<0.001*	47.6 ± 12.5	49.1 ± 10.0	0.196
Sex, *n* (%)			<0.001*			0.633
Male	164 (57.5)	370 (38.3)		108 (49.3)	113 (51.6)	
Female	121 (42.5)	597 (61.7)		111 (50.7)	106 (48.4)	
Weight (mean ± s.d.), kg	65.9 ± 13.9	62.0 ± 11.4	<0.001*	64.9 ± 13.2	64.6 ± 12.3	0.880
Height (mean ± s.d.), m	1.65 ± 0.09	1.62 ± 0.09	<0.001*	1.64 ± 0.10	1.64 ± 0.09	0.712
BMI (mean ± s.d.), kg/m^2^	24.2 ± 4.4	23.6 ± 3.4	0.184	24.1 ± 4.2	24.0 ± 3.5	0.912
Current/ever smoker, *n* (%)			<0.001*			0.462
Yes	77 (27.0)	78 (9.1)		181 (82.6)	44 (20.1)	
No	208 (73.0)	778 (90.9)		38 (17.4)	175 (79.9)	
Current back pain, *n* (%)			<0.001*			0.775
Yes	258 (90.5)	325 (40.0)		192 (87.7)	190 (86.8)	
No	27 (9.5)	488 (60.0)		27 (12.3)	29 (13.2)	
Back pain age of onset (mean ± s.d.), years	32.2 ± 13.0	36.3 ± 10.8	<0.001*	34.2 ± 13.4	34.4 ± 10.8	0.331

* Statistically significant.

### Propensity score matching

Based on the patient characteristics in (Table 1), baseline covariates of age [3], sex [22], weight, height [23], smoking history [24] and presence of current back pain [25] were selected for the calculation of propensity scores, for each participant, for the propensity of having SpA with the logistic regression model.

**Figure 2.**
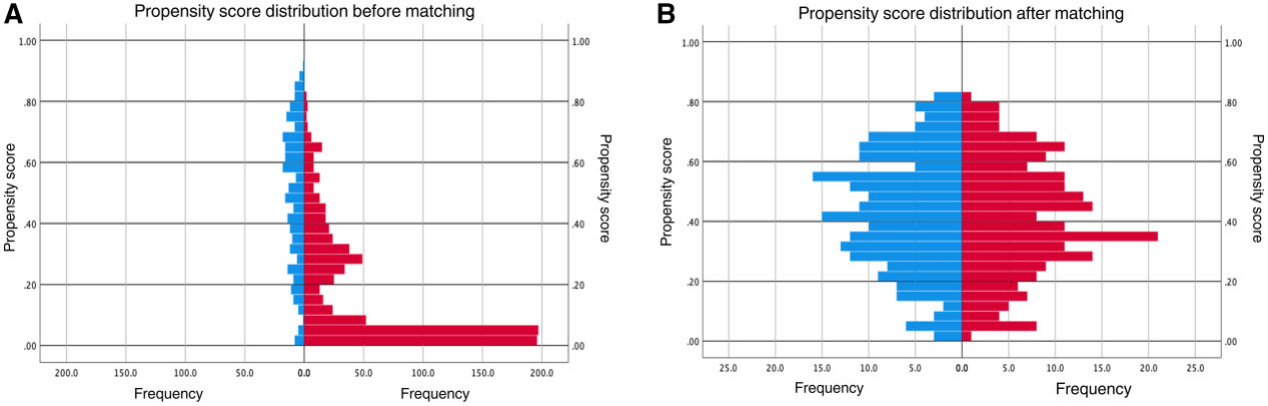
Propensity score frequency distribution before matching (**A**) and after matching (**B**)

A 1:1 propensity score by nearest neighbour matching was performed. The match tolerance of propensity scores determines the number of SpA participants that can be matched with the population participants. The smaller the tolerance, the fewer participants can be matched. A match tolerance of 0.1 s.d. was found to contain 237 participants in each group, but there were still significant differences (*P* = 0.005) between the two groups in terms of the propensity score after an initial trial matching. Therefore, the match tolerance was eventually set to 0.05 s.d. of the logit of the estimated propensity score to ensure matched pairs were within a narrower range. After matching, we checked the propensity score matching by evaluating the propensity scores (no significant difference between groups, *P* = 0.418; standardized differences, Cohen’s *d *=* *0.079; ratio of variances of propensity scores was 0.93), and the balance of all observed covariate across the two groups was shown by the standardized mean difference (Cohen’s *d* values of <0.25). This included 219 participants in each group in the comparative analysis of severity of DD. The distribution of propensity scores is shown in Fig. 2.

The severity of DD in each segment was assessed using the overall sum of Pfirrmann scores in each segment. Cervical segment refers to the group of discovertebral units that spans from C2/3 to C7/T1, Thoracic segments from T1/2 to T12/L1 and lumbar segments from L1/2 to L5/S1. By using the Mann–Whitney *U* test, overall Pfirrmann scores and disc herniation scores were compared between the two groups. The χ^2^ test was used to detect any difference in prevalence of high-intensity zone lesions, Schmorl’s node and Modic change in each segment, in addition to DD per disc level. For the sub-analysis of the SpA cohort, a multivariable stepwise logistic regression was used to detect any association between disease activity categories and the presence of DD, with adjustment for age, sex and BMI in the first step. In order to ascertain the difference in determinants of DD in early *vs* later disease, participants were stratified into a group with early disease, defined as symptom onset within 3 years, and a group with later disease, defined as symptom onset >3 years ago [26–28]. Symptom onset refers to the start of inflammatory back pain, satisfying either Caitlin or Berlin criteria [29].

Pearson’s correlation coefficient (*r*) was used to demonstrate the strength of any correlations between SpA clinical assessment, imaging and demographics. Univariate linear regression was then used to detect factors associated with a worse lumbar Pfirrmann score. Variables with a *P*-value of <0.05 were then included in a multivariable regression model, with age, sex and BMI adjusted in the first step. We used multivariable stepwise regression models owing to the exploratory nature of the research, in order to assess the variance in DD explained by variables such as disease activity and structural changes, above and beyond demographic characteristics such as older age and higher BMI. Odd ratios (ORs) and 95% CIs were reported as appropriate.

Cronbach’s α was used to assess intra- and inter-observer reliabilities for the DD-related parameters. An α value of 0.90–1.00 was noted to have excellent reliability, and an α value of 0.80–0.89 was noted to have good reliability [30]. All images were read and graded by two readers, with inter-observer agreement first tested using 20 participants randomly drawn from the study population by a third person (a statistician). The same 20 images were read again in a randomized order after 4 weeks of the first reading. Intra-observer reliability was noted to be good to excellent (α = 0.84–0.93), as was inter-observer reliability (α = 0.89–1.00) between two independent observers. The intraclass correlation coefficient was used to determine the inter-reader agreements of the SPARCC spine and SI joint MRI indexes. The agreement was interpreted as slight (0.00–0.20), fair (0.21–0.40), moderate (0.41–0.60), substantial (0.61–0.80) or almost perfect (0.81–1.00). The intraclass correlation coefficients of SPARCC SI joint MRI score and SPARCC spine MRI score were 0.88 and 0.79, respectively, indicating that the agreements between two readers were substantial to almost perfect.

Statistical tests were carried out using SPSS v.27.0 software (SPSS Inc.), and a *P*-value of <0.05 was treated as significant.

## Results

A total of 1271 individuals were enrolled into this study (76.1% population and 23.9% SpA), with 42.9% (545) males and 57.1% (726) females. A total of 219 matched pairs were identified, with 438 participants in total from both cohorts. In the matched cohort, all demographic characteristics had no significant difference between the two groups (Table 1).

The SpA patients had a better overall Pfirrmann score in the cervical (*P* < 0.001), thoracic (*P* < 0.001) and lumbar (*P* < 0.001) segments (Table 2). In the by-level analysis, levels having the most significant difference (where *P* < 0.001) between the two cohorts were T5/6, T6/7, L4/5 and L5/S1 (Table 3; Supplementary Fig. S1, available at *Rheumatology Advances in Practice* online). Conversely, SpA patients had a higher prevalence of anterior marrow change in the cervical (49.8% *vs* 20.5%, *P* < 0.001), thoracic (53.0% *vs* 21.5%, *P* < 0.001) and lumbar (43.4% *vs* 30.6%, *P* = 0.004) segments, but a lower prevalence of overall Modic change in the cervical (23.7% *vs* 35.6%, *P* = 0.006) and lumbar (19.2% *vs* 30.6%, *P* = 0.006) segments.

**Table 2.** Comparison of overall disc degeneration phenotypes between cohorts, before and after matching

	Before matching			After matching		
Variable	SpA	Population	*P*-value	SpA	Population	*P*-value
Overall Pfirrmann score (mean ± s.d.)						
Cervical	18.6 ± 2.8	20.1 ± 2.3	<0.001*	19.0 ± 2.8	20.0 ± 2.5	<0.001*
Thoracic	28.8 ± 5.8	34.9 ± 6.6	<0.001*	29.8 ± 6.1	33.3 ± 7.2	<0.001*
(Upper)	9.8 ± 2.3	11.9 ± 2.4	<0.001*	10.2 ± 2.4	11.4 ± 2.6	<0.001*
(Middle)	9.8 ± 2.4	12.3 ± 2.7	<0.001*	10.2 ± 2.5	11.6 ± 3.1	<0.001*
(Lower)	9.2 ± 1.8	10.7 ± 2.2	<0.001*	9.4 ± 1.9	10.2 ± 2.2	<0.001*
Lumbar	12.4 ± 2.6	14.9 ± 2.8	<0.001*	12.8 ± 2.7	14.7 ± 2.8	<0.001*
Overall herniation score (mean ± s.d.)						
Cervical (anterior)	1.17 ± 1.63	1.24 ± 1.67	0.397	1.2 ± 1.6	1.1 ± 1.6	0.367
Cervical (posterior)	1.65 ± 1.74	2.58 ± 1.84	<0.001*	1.8 ± 1.7	2.6 ± 1.8	<0.001*
Thoracic (anterior)	0.49 ± 1.43	0.67 ± 1.33	<0.001*	0.5 ± 1.3	0.7 ± 1.3	0.007*
Thoracic (posterior)	0.09 ± 0.492	0.35 ± 0.94	<0.001*	0.1 ± 0.5	0.3 ± 0.8	<0.001*
Lumbar (anterior)	0.58 ± 1.40	1.00 ± 1.56	<0.001*	0.7 ± 1.6	1.1 ± 1.7	<0.001*
Lumbar (posterior)	0.53 ± 1.06	1.85 ± 1.77	<0.001*	0.6 ± 1.2	2.0 ± 1.8	<0.001*
High-intensity zone lesion, *n* (%)						
Cervical	69 (22.7)	180 (18.6)	0.118	51 (23.3)	39 (17.8)	0.163
Thoracic	19 (6.3)	285 (93.8)	0.023*	15 (6.8)	7 (3.2)	0.08
Lumbar	98 (32.2)	399 (41.3)	0.005*	78 (35.6)	83 (37.9)	0.62
Schmorl’s node, *n* (%)						
Cervical	72 (7.4)	72 (7.4)	0.191	14 (6.4)	17 (7.8)	0.567
Thoracic	33 (10.9)	92 (9.5)	0.493	23 (10.5)	22 (10.0)	0.875
Lumbar	57 (18.8)	195 (20.2)	0.584	43 (19.6)	41 (18.7)	0.808
Anterior marrow change, *n* (%)						
Cervical	151 (49.7)	199 (20.6)	<0.001*	109 (49.8)	45 (20.5)	<0.001*
Thoracic	157 (51.6)	228 (23.6)	<0.001*	116 (53.0)	47 (21.5)	<0.001*
Lumbar	126 (41.4)	260 (26.9)	<0.001*	95 (43.4)	66 (30.6)	0.004*
Overall Modic change, *n* (%)						
Cervical	70 (23.0)	370 (38.3)	<0.001*	52 (23.7)	78 (35.6)	0.006*
Thoracic	45 (14.8)	108 (11.2)	0.091	30 (13.7)	21 (9.6)	0.18
Lumbar	59 (19.4)	260 (26.9)	0.008*	42 (19.2)	67 (30.6)	0.006*

Comparison of overall Pfirrmann scores and disc herniation scores (using Mann–Whitney *U* test) and high-intensity zone lesions, Schmorl’s node, anterior marrow change and Modic change in cervical, thoracic and lumbar segments (using χ^2^ test) before and after matching.

* Statistically significant.

**Table 3.** Results of χ^2^ test for by-level differences in disc degeneration prevalence between SpA cohort and population cohort

DD by disc level	SpA [*n* (%)]	Population [*n* (%)]	*P*-value
C2/3 DD	123 (56.2)	89 (40.6)	0.001*
C3/4 DD	108 (49.3)	101 (46.1)	0.503
C4/5 DD	84 (38.4)	95 (43.4)	0.285
C5/6 DD	76 (34.7)	111 (50.9)	0.001*
C6/7 DD	46 (21.0)	74 (33.9)	0.002*
C7/T1 DD	22 (10.0)	30 (13.7)	0.237
T1/2 DD	27 (12.3)	45 (20.5)	0.02*
T2/3 DD	21 (9.6)	47 (21.5)	0.001*
T3/4 DD	23 (10.5)	41 (18.7)	0.015*
T4/5 DD	22 (10.0)	37 (16.9)	0.036*
T5/6 DD	24 (11.0)	54 (24.7)	<0.001*
T6/7 DD	33 (15.1)	66 (30.3)	<0.001*
T7/8 DD	34 (15.5)	59 (26.9)	0.003*
T8/9 DD	27 (12.3)	50 (22.9)	0.004*
T9/10 DD	24 (11.0)	34 (15.5)	0.159
T10/11 DD	15 (6.8)	18 (8.2)	0.587
T11/12 DD	7 (3.2)	12 (5.5)	0.241
T12/L1 DD	7 (3.2)	5 (2.3)	0.558
L1/2 DD	8 (3.7)	20 (9.1)	0.019*
L2/3 DD	22 (10.0)	38 (17.4)	0.026*
L3/4 DD	36 (16.4)	54 (24.7)	0.033*
L4/5 DD	51 (23.3)	97 (44.3)	<0.001*
L5/S1 DD	51 (23.3)	97 (44.3)	<0.001*

DD: disc degeneration.

* Statistically significant.

Within the SpA cohort, participants with high or very high disease activity (*n* = 180), as defined by ASDAS-CRP, compared with the reference of inactive or lower disease state (*n* = 101) was not associated with presence of DD in the cervical segment (B = 1.497, *r*^2^ = 0.014, *P* = 0.166), thoracic segment (B = 0.753, *r*^2^ = −0.003, *P* = 0.953), lumbar segment (B = 0.829, *r*^2^<0.001, *P* = 0.569) and in the whole spine (B = 1.017, *r*^2^ < 0.001, *P* = 0.953).

Upon stratification of SpA patients into early disease (≤3 years) and late disease (>3 years), it was noted that patients with late disease had significantly higher mSASSS, SPARCC spine MRI index and BASMI scores (Supplementary Fig. S2, available at *Rheumatology Advances in Practice* online). In addition, males had higher SPARCC scores (*P* < 0.001) and mSASSS (*P* < 0.001) than females (Supplementary Table S2, available at *Rheumatology Advances in Practice* online). Pearson’s correlation test showed that mSASSS (*r *=* *0.431, *P* < 0.001) and BASMI (*r *=* *0.479, *P* < 0.001) were associated with age, and mSASSS and BASMI had a strong positive correlation (*r *=* *0.630, *P* < 0.001). In univariate linear regression (shown in Supplementary Table S3, available at *Rheumatology Advances in Practice* online), mSASSS (β = 0.131, *P* = 0.015), SPARCC spine MRI index (β = 0.129, *P* = 0.018) and BASMI (β = 0.252, *P* < 0.001) were associated with worse lumbar Pfirrmann scores. After adjustment for age (β = 0.549, *P* < 0.001), sex (β = −0.101, *P* = 0.066) and BMI (β = 0.076, *P* = 0.145), only SPARCC spine MRI index (β = 0.138, *P* = 0.010) was associated with a worse lumbar Pfirrmann score, whereas mSASSS (*P* = 0.119) and BASMI (*P* = 0.422) lost their significance. However, upon stratification into early and later disease with the adjustment of age, sex, BMI and the number of lumbar anterior marrow changes (Table 4), SPARCC spine MRI index was associated with higher lumbar Pfirrmann scores in early disease (β = 0.260, *P* = 0.023), and mSASSS was associated with lower lumbar Pfirrmann scores in later disease (β = −0.200, *P* = 0.018).

**Table 4.** Multivariable linear regression for association between spinal inflammation, structural changes and loss of spinal mobility with lumbar Pfirrmann score

	Early (back pain duration ≤3 years)				Later (back pain duration >3 years)			
Variable	Regression coefficient (B)	Standard coefficient (β)	*R* ^2^	*P*-value	Regression coefficient (B)	Standard coefficient (β)	*R* ^2^	*P*-value
Age	0.082	0.628	0.351	<0.001*	0.097	0.517	0.289	<0.001*
Sex	0.105	0.029	0.006	0.772	−0.660	−0.144	0.037	0.030*
BMI	0.058	0.150	0.047	0.125	0.027	0.050	0.025	0.431
Number of lumbar anterior marrow changes	−0.171	−0.107	0.041	0.379	−0.049	−0.034	0.001	0.629
mSASSS	0.027	0.134	0.387	0.283	−0.025	−0.200	0.326	0.018*
SPARCC spine MRI index	0.058	0.260	0.420	0.023*	0.032	0.119	0.328	0.073
BASMI	−0.222	−0.186	0.373	0.126	0.207	0.154	0.327	0.074

Multivariable linear regression for association between spinal inflammation (SPARCC spine), structural changes (mSASSS) and loss of spinal mobility (BASMI) with lumbar Pfirrmann score, after stratification into early (back pain duration ≤3 years) and later (back pain duration >3 years) disease.

* Statistically significant.

For the prediction of whole spine DD within the SpA cohort alone, males had lower odds of having whole spine DD than females [OR 0.622 (95% CI 0.419, 0.951); *P* = 0.028; Supplementary Table S4, available at *Rheumatology Advances in Practice* online], whereas an older age was associated with higher odds of whole spine DD [OR 1.095 (95% CI 1.072, 1.119); *P* < 0.001]. For comparison, the result from the population cohort showed that the presence of whole spine DD was independent of patient’s sex (*P* = 0.109).

## Discussion

In this study, we found that SpA patients had an overall lower prevalence of DD as defined by the Pfirrmann score, and DD severity was less severe by segment and within the whole spine compared with the general population. The presence of DD was found to be independent of SpA disease activity based on the BASDAI and ASDAS. However, when we stratified patients into early disease and late disease based on their symptom onset, we discovered that SpA disease duration had different associations with DD. In patients with early disease, the SPARCC spine MRI index was positively associated with higher lumbar Pfirrmann scores. Males had higher mSASSS, and mSASSS was negatively associated with lumbar Pfirrmann scores. Female had higher odds of DD in the whole spine.

The pathophysiology of DD is complex and relies on the interplay between various factors, including mechanical, genetic, systemic and toxic factors, in addition to ageing. The positive association between age and DD can be easily understood and explained. The intervertebral discs are an integral part of the spinal column, contributing to its stability and flexibility. A normal disc consists of three basic structures, namely the cartilage endplate, the nucleus pulposus and the annulus fibrosus, and they contain collagen, proteoglycan and water synthesized by different cells, such as chondrocyte-like cells and fibroblast-like cells [31, 32]. However, the constituents of the matrix do not remain unchanged, and they are continuously being degraded by enzymes, the MMPs, which are secreted by chondrocytes. Cytokines, such as IL-1, interferon andTNF-α, have been shown to inhibit the synthesis of the matrix and promote the production of MMPs, and this may explain why patients with a higher degree of inflammation can have a higher risk of DD.

Physiologically, the levels of water and glycoprotein in the discs decrease with ageing, leading to fibrosis and calcifications. In addition, numerous other factors, such as mechanical stress, trauma, smoking, immobilization, vascular diseases and genetic factors, have been implicated in the aetiology of DD [33, 34]. Studies have also shown that the expression of matrix-degrading enzymes, especially MMPs, plays an important role in DD by degrading collagen and proteoglycans. The correlation between DD and spinal inflammation in SpA patients might be explained by the elevated levels of MMP3 in patients with AS [35]. Another study analysing the bony and soft tissue surgical specimens of patients with AS and degenerative disc disease found elevated cathepsin K and MMP1, which have been implicated in bone resorption and cartilage tissue destruction [35]. Furthermore, AS is associated with accelerated atherosclerosis and increased risk of endothelial dysfunction, which may predispose AS patients with more spinal inflammation to DD, because discs rely on transport capacity through the capillary wall for nutrients [34, 36]. The SPARCC MRI inflammation index also co-incides with Modic type I changes, both of which are seen when fissuring of the endplates with the development of vascular granulation tissue adjacent to them results in bone marrow oedema [13]. We believe the endplate marrow oedema seen on MRI could well be reflecting the degree of inflammation within the disc, because the SPARCC MRI index was positively associated with higher lumbar Pfirrmann scores. Whether the inflammation score SPARCC is a prognostic factor for deteriorating Pfirrmann scores should be verified in further studies. Nevertheless, all of the above factors explain the positive association between active spinal inflammation in SpA patients with early disease and DD. Although similar findings were not reciprocated using BASDAI and ASDAS, it is known that ASDAS and particularly BASDAI are not well correlated with active inflammation on MRI, hence they might not reflect the inflammatory activity [37].

In patients with later (>3 years in duration) disease, mSASSS was negatively associated with lumbar Pfirrmann scores after adjustment for demographic confounding factors. mSASSS is the most frequently used and validated method for quantifying spinal radiographic structural changes, including erosions, sclerosis and syndesmophytes, in SpA patients, and a higher mSASSS indicates the accrual of more chronic structural damage. Previous studies have shown that patients with longer disease duration tend to have more syndesmophytes and higher mSASSSs [38, 39], and male SpA patients have a higher prevalence of bamboo spine [40] and ankyloses than females [41]. One study found that the *ANKH* gene variant encoding for progressive ankylosing protein was associated with SpA in males [42, 43]. Conversely, females report a worse patient-reported disease activity outcome, such as BASDI and ASDAS [44]. In addition, females also experience more delay in diagnosis than males [45], and this can be attributable to the earlier radiographic damage (mSASSS) [44] and ankylosis [46] in males. Hence, we believe that a higher degree of ankylosis in male SpA patients confers a protective effect for DD, because their spine is relatively more stiff and will experience less spinal motion [47] and subsequent axial loading. The relationship between segmental instability and DD has also been noted in models of spinal fusion, where compensatory increase in motion, instability in adjacent levels [48], short fusion segment and a high BMI [49] were found to cause adjacent segment degeneration. This might explain why SpA patients with a longer disease duration and higher mSASSS, which in our cohort were found in males rather than females, have less DD, because a higher degree of ankylosis means the spine is more stable, hence these patients do not experience as much instability, increase in motion or axial loading compared with patients with less ankylosis.

Disc levels in which the SpA cohort had the most significantly lower prevalence of DD included T5/6, T6/7, L4/5 and L5/S1 levels (*P* < 0.00), coinciding with what was found in a populational whole spine DD prevalence report by Teraguchi *et al.* [19], in which disc levels C5/6, T6/7 and L4/5 had the highest prevalence of DD. The evidence suggests that the disc-protective effect of SpA is most evident at levels with the highest propensity to degenerate. It is noteworthy that the cervical spine has comparatively less SpA disease involvement than the thoracic and lumbar spine [50]. This can be explained, in part, by results of an alignment study, in which Lee *et al.* [51] found the most common horizontal thoracic level to be the T7 body and the most common horizontal lumbar level to be the L4 body, in addition to a postural loading study, in which Keller *et al.* [52] found compressive stress to be highest in the mid-thoracic region and shear stress to be highest in the L5–S1 region. We also found that the prevalence of Modic changes was significantly higher in the population cohort compared with the SpA patients. Our study results echoe some of the findings previously reported by de Bruin *et al.* [8], based on the DESIR cohort, in which Modic changes were found more often in patients with chronic back pain compared with patients with axial SpA. Moreover, SpA patients with early disease appear to have more DD, owing to increased inflammatory activity and endothelial dysfunction, as illustrated above. In patients with longer disease duration and established radiographic damage, the presence of a higher degree of ankylosis and less mobility might confer a protective effect against DD owing to greater stability and less axial loading. Further studies are warranted to confirm these hypotheses and shed light on the underlying pathophysiology.

The strength of this study comes from the direct comparison between two cohorts for DD severity, in which the matched groups were sizeable (219 participants each). However, there are limitations to note. Participants in the two cohorts were recruited by two different recruitment protocols in different centres and rheumatology clinics. Before matching, SpA patients were younger, more likely to be male and smokers, and had higher body weight and height. Therefore, the severity of DD was subjected to these confounding factors. Nevertheless, after propensity score matching, all demographic factors, including age (*P* = 0.196), revealed no significant differences. However, we should note that there could still be factors not adjusted between two cohorts, which, for example, includes the occupation and physical activity of each participant.

In summary, through propensity score-matched analysis between two longitudinal cohorts, we found a lower severity of DD in SpA patients compared with the general population, which was especially noticeable at disc levels traditionally considered to have the highest susceptibility for DD (T5/6, T7/8, L4/5 and L5/S1 levels). The SPARCC spine MRI index was associated with higher lumbar Pfirrmann scores in early disease, whereas the mSASSS was associated with lower lumbar Pfirrmann scores in later disease. We found that older, female SpA patients had higher odds of having DD than male SpA patients. Overall, SpA patients have less severe DD, especially for men and those with later disease and a higher mSASSS. These findings should raise the awareness of the attending physicians when encountering SpA patients with back pain, and timely referrals and appropriate investigations should be instigated when necessary.

## Supplementary Material

rkae015_Supplementary_Data

## Data Availability

The data supporting the findings of this study are available within the article and its supplementary materials.
