# Supplementary material for: Male spondyloarthritis patients and those with longer disease duration have less severe disc degeneration: propensity score-matched comparison
Source: Rheumatol Adv Pract. 2024 Feb 6;8(1):rkae015. doi: 10.1093/rap/rkae015 (PMC10884529; doi:10.1093/rap/rkae015)
Supplement: rkae015_Supplementary_Data [file rkae015_supplementary_data.docx]

| Demographics and imaging parameter | Mean ± S.D. (range) |
| --- | --- |
| Age | 44.89 ± 12.96 (18-80) |
| Sex | Female: 42.2 %; Male: 57.8% |
| Weight (kg) | 65.82 ± 13.73 (38-111) |
| Height (m) | 164.68 ± 9.41 (136-198) |
| BMI | 24.21 ± 4.38 (15.05-38.70) |
| Backpain duration (n=286) | 12.46 ± 11.43 (0.0-49.0) |
| Age of onset of backpain (n=286) | 32.34 ± 12.97 (8-69) |
| BASMI (n=282) | 3.53 ± 1.69 (0.42-8.82) |
| SPARCC (n=268) | 6.22 ± 8.51 (0.0-40.5) |
| mSASSS (n=276) | 10.60 ± 17.06 (0.0-72.0) |
| Family history & disease activity | Percentage (number) |
| Family history of SpA (n=277) | 21.7% (60) |
| Ever had uveitis (n=286) | 29.4% (84) |
| Ever had Crohn's disease (n=286) | 0.7% (2) |
| Ever had Ulcerative colitis (n=286) | 1.7% (5) |
| Ever had IBD (n=287) | 3.1% (9) |
| Ever had Psoriasis (n=287) | 15.3% (44) |
| Ever had Peripheral Arthritis (n=287) | 55.7% (160) |
| Ever had enthesitis (n=285) | 44.9% (128) |
| Current dactylitis (n=286) | 8.0% (23) |
| ASDAS (CRP) disease activity state | Percentage (number) |
| Inactive | 11.7% (33) |
| Low | 24.2% (68) |
| High | 41.3% (116) |
| Very high | 22.8% (64) |

Supplementary Table S1. Patient characteristics for Spondyloarthritis subcohort analysis (n= 287)

Supplementary Table S2. Mann-Whitney *U* test for gender differences of spinal inflammation, mobility, and structural change

| Variable (means ± S.D.) | Male | Female | p-value |
| --- | --- | --- | --- |
| BASMI | 3.59 ± 1.68 | 3.30 ± 1.66 | 0.08 |
| SPARCC spine MRI index | 7.76 ± 9.25 | 3.50 ± 5.84 | <0.001* |
| mSASSS | 13.43 ± 19.30 | 5.73 ± 11.40 | <0.001* |
| ASDAS_CRP | 2.67 ± 1.10 | 2.46 ± 1.14 | 0.053 |
| ASDAS_ESR | 2.85 ± 0.99 | 3.12 ± 1.03 | 0.017* |

*tested with independent sample t-test

Supplementary Table S3. Univairate and multivariable linear regression for association between spinal inflammation (SPARCC spine), structural changes (mSASSS), and loss of spinal mobility (BASMI) with lumbar Pfirrmann score

| Variable | Univariate linear regression | | |  | Multivariable linear regression | | |
| --- | --- | --- | --- | --- | --- | --- | --- |
|  | Regression coefficient (B) | Standardised coefficient (β) | R^2^ | p-value | Regression coefficient (B) | Standardised coefficient (β) | p-value |
| Age | 0.086 | 0.487 | 0.24 | <0.001* | 0.099 | 0.595 | <0.001* |
| Male sex | -0.496 | -0.107 | 0.011 | 0.04* | -0.438 | -0.101 | 0.066 |
| BMI | 0.08 | 0.156 | 0.02 | 0.006* | 0.038 | 0.076 | 0.145 |
| Back pain duration | 0.023 | 0.011 | 0.013 | 0.028* | -0.021 | -0.11 | 0.057 |
| Smoker | -0.183 | -0.036 | 0.001 | 0.494 |  |  |  |
| Drinker | -0.062 | -0.008 | 0 | 0.877 |  |  |  |
| HLA-B27 positivity | -0.361 | -0.063 | 0.004 | 0.239 |  |  |  |
| Family history of SpA | 0.343 | 0.062 | 0.004 | 0.242 |  |  |  |
| Radiographic axial SpA | 0.314 | 0.042 | 0.002 | 0.448 |  |  |  |
| mSASSS | 0.017 | 0.131 | 0.017 | 0.015* | -0.012 | -0.092 | 0.119 |
| On sulfasalazine | 0.075 | 0.014 | 0 | 0.788 |  |  |  |
| SPARCC spine MRI index | 0.033 | 0.129 | 0.017 | 0.018* | 0.035 | 0.138 | 0.010* |
| BASMI | 0.344 | 0.252 | 0.064 | <0.001* | 0.075 | 0.058 | 0.422 |
| ASDAS_CRP | -0.086 | -0.042 | 0.002 | 0.427 |  |  |  |
| ASDAS_ESR | -0.118 | -0.067 | 0.005 | 0.172 |  |  |  |

*BMI* body mass index, *HLA-B27* human leukocyte antigen B27, *SPARCC* spine Spondyloarthritis Research Consortium of Canada spine MRI index, *mSASSS* modified Stoke Ankylosing Spondylitis Spine Score, *BASMI* Bath Ankylosing Spondylitis Metrology Index, *ASDAS* Ankylosing Spondylitis Disease Activity Score, *CRP* C-reactive protein, *ESR* Erythrocyte sedimentation rate

Supplementary Table S4. Univariate logistic regression for the prediction of DD across whole spine

|  | Odds ratio (95% CI) | p-value |  |
| --- | --- | --- | --- |
| Age | 1.095 (1.072-1.119) | <0.001* |  |
| Sex (Male) | 0.622 (0.419-0.951) | 0.028* |  |
| BMI | - | 0.326 |  |
| Underweight | 0.509 (0.184-1.405) | 0.193 |  |
| Overweight | 1.200 (0.703-2.047) | 0.504 |  |
| Obese | 1.312 (0.708-2.431) | 0.388 |  |
| Current back pain | 0.828 (0.407-1.683) | 0.602 |  |
| Early back pain < 3 years | 0.521 (0.327-0.832) | 0.006* | (confounded by age) |
| Backpain Age <40 | 0.270 (0.156-0.467) | <0.001* | (confounded by age) |
| Smoker | 1.211 (0.760-1.929) | 0.421 |  |
| Drinker | 0.617 (0.309-1.234) | 0.173 |  |
